# Animal Assisted Activities (AAAs) with Dogs in a Dialysis Center in Southern Italy: Evaluation of Serotonin and Oxytocin Values in Involved Patients

**DOI:** 10.3390/biomedicines13122944

**Published:** 2025-11-29

**Authors:** Antonio Santaniello, Giuseppe Perruolo, Alessia Amato, Susanne Garzillo, Federica Mormone, Cristina Morelli, Pietro Formisano, Mario Sansone, Alessandro Fioretti, Francesco Oriente

**Affiliations:** 1 Department of Veterinary Medicine and Animal Production, University of Naples Federico II, 80137 Naples, Italy; alessiaamatovet@gmail.com (A.A.); susannegarzillo@gmail.com (S.G.); fioretti@unina.it (A.F.); 2 Department of Translational Medicine, University of Naples Federico II, 80138 Naples, Italy; giuseppe.perruolo@unina.it (G.P.); federica.mormone@unina.it (F.M.); cristina.morelli@unina.it (C.M.); pietro.formisano@unina.it (P.F.); francesco.oriente@unina.it (F.O.); 3 Department of Electrical Engineering and Information Technologies, University of Naples Federico II, 80125 Naples, Italy; mario.sansone@unina.it

**Keywords:** animal-assisted activities (AAAs), dog, human–animal relationship, serotonin, oxytocin, dialysis, one health

## Abstract

**Background/Objectives**: In the present study, the changes in oxytocin (OXT) and serotonin (5-HT), as hormones involved in social relationships and mood regulation, respectively, were measured in dialysis patients involved in Animal Assisted Activity (AAA) interventions. **Methods**: Thirty patients (15 men and 15 women) with chronic kidney disease, undergoing hemodialysis three times per week, for 4 h, were enrolled. The patients were divided into three groups: two experimental groups who received the AAA intervention and a control group that never received the AAA intervention. A specific dog-zootherapist vet pair was assigned for each experimental group. All sessions of the two experimental groups were performed weekly, for a total period of 3 months (12 sessions). Blood samples were collected at the beginning and end of each session (T_0_ and T_1_), lasting about one hour. The interaction time with the dog was approximately 40 min. The samples were then analyzed to measure the levels of oxytocin and serotonin and processed using analysis of variance with mixed effects models. **Results**: The results obtained showed that both dog-zootherapist vet dyads caused a statistically significant overall effect of both oxytocin and serotonin, increasing during the sessions, compared to the control group. In addition, it was observed progressively increasing effect between two consecutive weeks. **Conclusions**: The results from this study showed that the AAA represents a positive stimulus for patients on dialysis. Thus, our study suggests that structured AAA intervention in a hemodialysis center can improve patients’ quality of life during the dialysis cycle.

## 1. Introduction

According to the One Health concept, human health and the environment are closely linked and influenced by multiple factors. This principle integrates different health problems through a multidisciplinary approach, emphasizing health promotion, based on the concept that human health is derived from the health of ecosystems [1].

The AAIs (Animal Assisted Interventions), as an example of the approach stated above, are interventions that involve the animal and aim at improving the health and well-being of people. They can be applied to patients of any age and affected by different pathologies and can be combined with traditional care, treatments, and social health interventions already in progress [2].

From the data reported in the literature, a relationship with animals improves the state of health in humans [2,3]. The effects of the AAIs, highlighted by scientific studies, range from the reduction in depression and anxiety to the stimulation of social and cognitive skills [4].

Several studies have dealt with the beneficial effects of AAI with dogs on human health, in particular on the results obtained in various physiological and psychological fields [3]. The research focused on the benefits brought in various contexts, such as the hospital environment [5,6], the school environment, the rehabilitation [7], Alzheimer’s disease [8], and many others. Nevertheless, very little research has been carried out in the field of hemodialysis.

This treatment is mainly used in patients with chronic renal failure, a disease that involves the progressive and irreversible loss of renal function. In addition, in such circumstances, hemodialysis represents the only solution to keep the patient alive.

Numerous studies can be found in scientific literature that investigate the affective and emotional dimensions in nephropathic patients [9,10,11,12,13].

These studies show a high presence of psychological discomfort, such as anxiety, stress, and depression, which influence the quality of life of the patients themselves [12].

Such effects may be related simply to routine hemodialysis treatment, which can often be stressful and painful [11]. In particular, especially in the initial phase of treatment, patients may present a lowering of mood due to changes in their physical condition and their social role [13].

In this case, the task of the AAIs could be to support patients in facing their treatment.

Another problem that should not be underestimated in patients undergoing hemodialysis is depression. Indeed, this psychological disorder has been found in patients at a percentage ranging from 20 to 30% [10].

In this regard, some studies have underlined that those psychological symptoms in dialysis patients have the same weight as physical symptoms and that, especially depressive symptoms, can compromise the clinical outcomes of renal disease and can double the possibility of not adhering to treatment [14].

Based on these considerations and on the importance that psychological factors have during renal disease, it is important to recognize that patients need help in dealing with the emergency and urgency of this condition, so that they can have support that helps them accept the presence of a chronic disease and adapt to the changes it brings [9].

In the recent literature, many studies have been conducted to scientifically support the physiological parameters measured during AAIs, to corroborate the positive results obtained [8].

In some of this research, the effects produced by AAA interventions have been measured through serum dosages of oxytocin (OXT) and serotonin (5-HT) [15].

Indeed, numerous studies corroborate the benefits given by the relationship with the dog linked to the modifications of oxytocin and serotonin [15,16,17].

In particular, some research has shown that oxytocin increases in the blood and urine of dogs and humans during their interaction [18].

Even harmless sensory stimulation, such as tactile interaction between dogs and humans, can increase oxytocin concentrations in both dogs and humans [19].

Oxytocin, therefore, is strongly implicated in the formation of affiliation bonds and influences trust, generosity, and group cooperation by modulating emotion recognition, empathy, and social perception [20].

Finally, some studies suggest that oxytocin also affects serotonergic function, thus stimulating the release of serotonin [21].

Despite the scarcity of studies, existing research has begun to examine the effects of AAIs in hemodialysis populations, particularly focusing on their potential modulation of oxytocin and serotonin pathways. However, the evidence remains limited, highlighting the need for further investigation in this area. Their results support the thesis that the relationship with the dog allows for the consolidation of the emotional relationship with the patient, positively influencing the mood and some hormonal parameters.

Based on these premises, the objective of this study was to investigate variations in oxytocin and serotonin levels among dialysis patients before and after AAA interventions.

## 2. Materials and Methods

### 2.1. Experimental Design and Patients

This randomized, controlled trial with 3 parallel groups has been approved by the Ethics committee of Federico II University of Naples-Naples (No.188/19 of 5 June 2019), and it adheres to the Declaration of Helsinki.

For this study, recruitment, screening, and treatment were carried out from April 2022 to June 2022. All participants were selected by the “Kidney Center” Physician and Psychologist and were required to submit written informed consent before randomization.

A total of 30 patients (15 men and 15 women) with an average age of 42.2 ± 7.57 (SD) years were enrolled in the study with comparable stages of renal impairment and relationship difficulties. The patients were divided into three groups, randomly stratified on sex: two experimental groups that performed the AAAs, each with a different dog-veterinary zootherapist dyad (VET1 and VET2), and a control group that did not perform the activity with the dog (CTRL). For all groups, the considered inclusion criteria were the following: medical history (function of both kidneys less than 70%; altered renal parameters as creatinine, approximately 8–11 mg/dL and blood urea nitrogen 40–150 mg/dL (threshold values in human: creatinine: 1.1–1.2 mg/dL, blood urea nitrogen: 20–30 mg/dL); dialytic treatment cycle for 3 times a week for 3 h per session; similar socio-demographic characteristics (i.e., years of education, cultural level, family background, not owning a dog, analogs life-style). Instead, the following exclusion criteria were considered: refusal to participate in the project, other levels of renal impairment, and use of antidepressants or mood-regulating medications. For the groups participating in the AAAs, further exclusion criteria were allergy to animals, dog phobia, and pet ownership.

**Figure 1:**
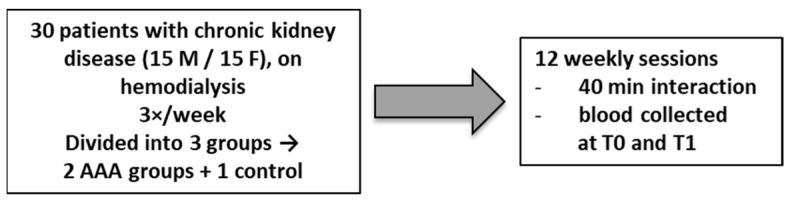
Sequential workflow and key steps of the study.

Before the start of the study, all patients provided and signed the informed consent form, which was drafted in accordance with ISO 9001-2015 Cert. n. 40593/20/S-26 [22] (Figure 1).

### 2.2. Methodology

The 30 patients in this study were divided into 3 groups: Experimental group 1 (VET1) with 10 patients (5 males and 5 females) received 1 AAA session per week lasting approximately 1 h, for a total of 12 sessions; experimental group 2 (VET2) with 10 patients (5 males and 5 females) received 1 AAA session per week lasting approximately 1 h, for a total of 12 sessions. The CTRL group with 10 patients (5 males and 5 females) makes up the control group and has never received AAA sessions, but other activities such as TV, listening to music, and reading.

The preliminary step of the AAA lasting 10 min involved the structuring of the setting and the initial greetings through the presentation of the dog-veterinary zootherapist dyad to patients, in the second phase lasting 40 min a structured activity through the game with the dog and an interview with the patients, in the final phase lasting 10 min a greeting ritual was carried out with oral discharge of the dog and hand washing.

### 2.3. AAA Team

The AAA team involved two veterinarians trained in accordance with interdisciplinarity and the interspecific relationship with their dogs [3]. The intervention was aimed at improving the quality of life of the patients during the dialysis cycle, favoring greater participation also in dialytic treatment.

### 2.4. Choice of the Dog

The species most capable of entering into a relationship with a man and activating his oxytocinergic system is the dog.

Particularly, due to the long process of co-evolution, man and dog have mutually modified each other, creating a basis for communication and mutual understanding. For this reason, many abilities of dogs, such as the capacity to read body language and facial expression, to perceive hormone secretions and different emotional states of humans, allow them to exhibit highly coordinated behavior with humans [23,24].

This predisposes the dog to a deep and complete relationship with AAI users. Furthermore, dogs and humans are particularly good at mutually activating the oxytocinergic system and generating oxytocin-related effects.

The dogs involved in this study were selected following Monash’s Canine Personality Test [25]. This test is useful for highlighting the dog’s high relational abilities and reliability [26]. The dogs were chosen after obtaining a good score based on cooperation motivation, extroversion, friendliness, and concentration [27]. In addition, co-therapist dogs were trained together with the zootherapist veterinarian, who is also the owner, following the Federico II Model of Healthcare Zooanthropology [3].

The dogs involved in this study were Sunday and Pongo.

Sunday is a Golden Retriever, male, aged 7 years old. He is a balanced and sensitive dog. He had optimal pedagogical development from an early age and has an enriched proximal level of experience. His positional profile presents an average arousal, a cheerful emotional structure, and the predominant motivations are affiliative, collaborative, and epimeletic.

Pongo is a Labrador Retriever, male, aged 9 years old. He is a dog naturally inclined to play and to form interspecific and intraspecific relationships. His emotional attitude is joyful, and his arousal is average. He is an extremely sensitive dog and capable of recognizing subtle emotional nuances.

### 2.5. Behavioral and Infectious Safety of the Dog

During the AAA intervention, all patients can interact with co-therapist animals by inter-specific relationship activities, providing brushing, playing, strolling, and then, potentially, contact with the dog’s mucosae and fur. In this context, patients can be exposed to bacteria, fungi, and parasites, also sub-clinically carried by the dog [28,29]. Particularly, as reported in the scientific literature, different and antibiotic-resistant bacterial species can be carried by the dog and transmitted to humans [8]. Therefore, the dogs involved in our study underwent monthly health checks throughout the study cycle. These conditions were also important for total patient safety from potential zoonotic risks [30].

In addition, at T_0_ and T_1_ of each AAA session, disinfectant wipes (chlorhexidine, TRIS-EDTA, zinc gluconate, and glycerin) were used to clean the coat, the fingertips, and the tail of the dog to avoid the transmission of zoonotic agents (e.g., bacteria, fungi, and parasitic elements).

### 2.6. Animal-Assisted Activities (AAAs)

AAAs were carried out from April 2022 to June 2022. They were held weekly for a total of 12 meetings, each lasting approximately 1 h, and were always conducted at the same time, during the second hour of the dialysis cycle. The access to the second hour allows operators to carry out connection operations to the dialysis machine without interference, just as the last hour is dedicated to removal operations.

In each session, in the initial 10 min, there was an introduction of the dog to patients; the next 20 min involved structured activity with the dog; a 20 min conversation with the patient; the last 10 min involved each time hand washing and final greetings. For the entire session, the dog remained an active part, whether it was actively participating in play [31] or resting conditions on the dog mat.

The presence of the dog in each session allowed the creation of a relational thrust between operator and patient and facilitated the establishment of trust. As can be seen from the present study, the therapeutic alliance relationship between the patient and the operator–dog couple takes place from the sixth to the seventh meeting according to a constant growth curve.

### 2.7. Blood Sampling and Analysis

We started the study by taking a blood sample from each patient during their last hour of dialysis. This allowed us to measure their initial or baseline levels of oxytocin and serotonin. For all 12 AAA sessions with the dog, as well as the last two without it, we collected blood samples before (T_0_) and immediately after (T_1_) each session. All samples were processed to obtain serum, which was then frozen until we were ready to test them for oxytocin and serotonin levels.

In detail, serum concentrations of oxytocin and serotonin were measured using two commercially available ELISA kits (Oxytocin ELISA Kit, Cat. No. ADI-900-153A; and Serotonin ELISA Kit, Cat. No. ADI-900-175; Enzo Life Sciences, Long Island, NY, USA) [32,33,34,35,36,37]. Both assays are for research use only (RUO) and employ a competitive colorimetric method. Patient serum was added to a microplate and incubated with serotonin or oxytocin conjugated with alkaline phosphatase for 2 h at room temperature. Next, the samples were further incubated with anti-serotonin or anti-oxytocin antibodies, and finally, pNpp was added to generate a colorimetric reaction, which was read in a photometer at a wavelength of 405 nm. All analyses were performed using the Triturus semi-automated ELISA processor (Grifols Movaco, Barcelona, Spain, Europe), which ensures precise reagent dispensing, incubation, and washing cycles. Each sample was analyzed in duplicate. The assays were conducted according to the manufacturer’s instructions, using 50 µL of serum per well. The assay sensitivity for oxytocin was 15 pg/mL (range 15–1000 pg/mL) and for serotonin 0.293 ng/mL (range 0.293–100 ng/mL). Data analysis was carried out using the proprietary Triturus software (ver. 4.01b), which applies a 4-parameter logistic curve-fitting (4PL) algorithm to interpolate sample concentrations from standard curves. Quality control was ensured by the inclusion of manufacturer-provided controls in each run. The coefficient of variation (CV) between duplicates was required to be <10% for sample validity.

### 2.8. Statistical Analysis

Due to the particular design of the study and to missing data, we used a specific repeated measures analysis to evaluate the effect of AAA treatment on the levels of serotonin and oxytocin at the beginning and the end of the session over the course of 12 weeks. In fact, it is known that repeated measures data do not satisfy independence assumptions required for standard ANOVA or linear regression, because a single subject undergoes multiple measurements thus introducing correlation among data points; moreover, because of missing data as showed in Figures S1 and S2 (34 observations out of 360) standard R-ANOVA (repeated measures ANOVA) is not adequate; therefore, in our study, linear mixed effects models (LME) were used [38]. As is known, LME can model fixed effects (in our case, ‘veterinarian’ and ‘week’) and random effects (in our study, ‘subject’): with this modeling, one can account for subject variability in response to the treatment. However, due to the particular nature of this method it is not possible to report standard results (regression coefficients, F-statistics, etc.) as in linear regression or ANOVA; instead Likelihood Ratio Test (LRT) is used to compare different models including or excluding interesting factors to establish the importance of each factor: in fact, the LRT gives a *p*-value which, if significant, indicates that the two compared models are different, indicating the importance of the factor excluded. For further discussion about LME, we refer the reader to [39,40].

Wherever required, a *p* < 0.05 has been considered significant.

The software used for LME analysis is R (ver. 4.4.3) [41]; in particular, we used the package LME4. We have treated ‘week’ and ‘veterinarian’ as fixed effects, while ‘patient’ has been treated as a random effect.

## 3. Results

**Figure 2:**
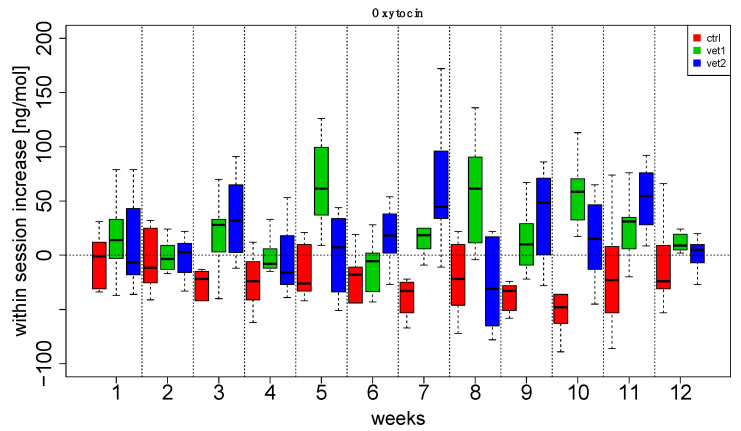
Oxytocin data aggregated by week and veterinarian. For each week, the distribution of patients’ increase has been reported vs. veterinary. Each boxplot summarizes 10 patients.

**Figure 3:**
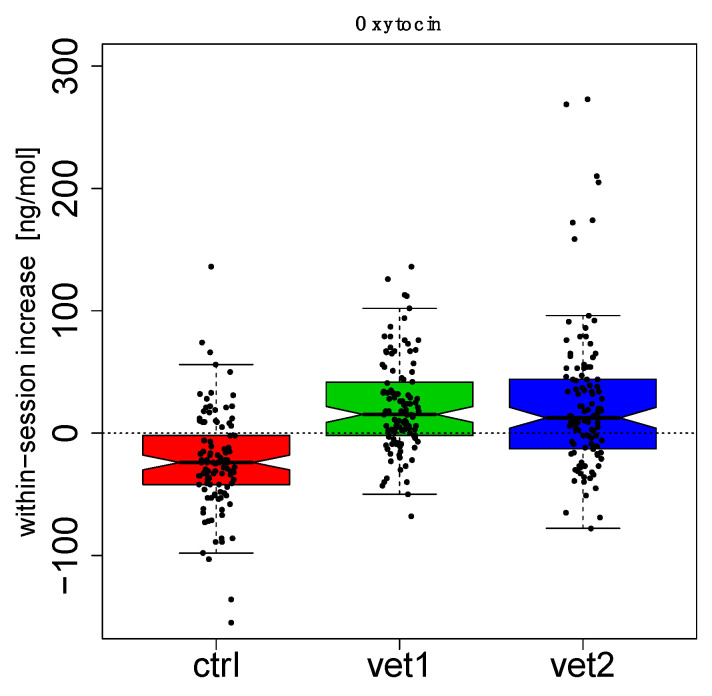
Oxytocin data aggregated by the veterinarian. For each VET, the distribution of within-session increase has been summarized with a boxplot. Dots represent single measures. Each boxplot summarizes 120 measures.

**Figure 4:**
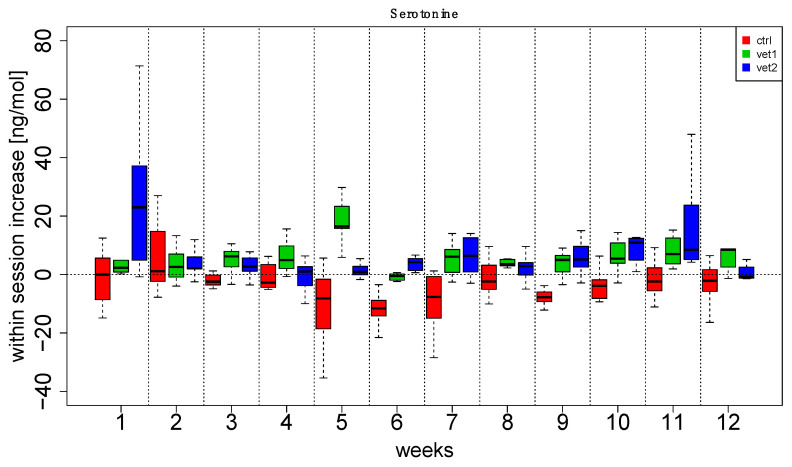
Serotonin data aggregated by week and veterinarian. For each week, the distribution of patients’ increase has been reported vs. veterinary. Each boxplot summarizes 10 patients’ measures.

**Figure 5:**
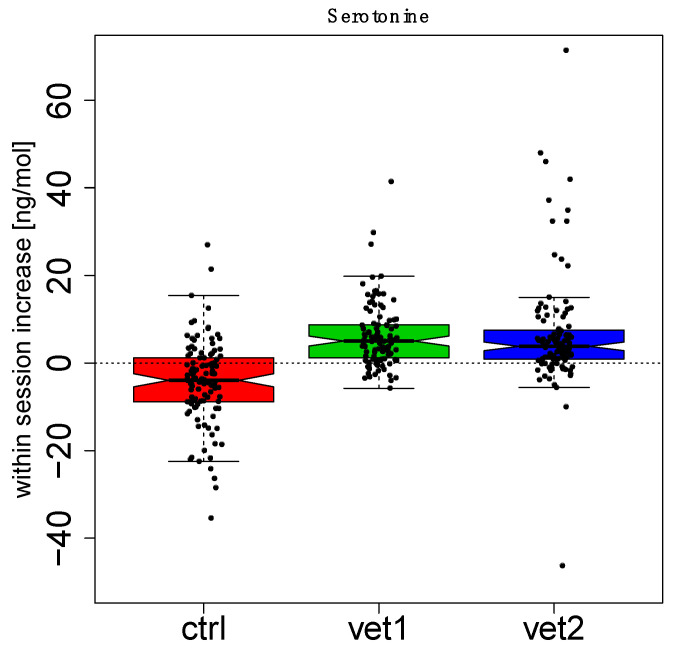
Serotonin data aggregated by the veterinarian. For each VET, the distribution of within-session increase has been summarized with a boxplot. Dots represent single measures. Each boxplot summarizes 120 measures.

Figure 2 and Figure 3 show boxplots (with the median in the center, interquartile boxes, and dashed-line whiskers for 1.5 Inter-Quartile-Range) of oxytocin within-session variation (between T0 and T1): data have been aggregated by the week session (Figure 2) and by the VET (Figure 3). Analogously, Figure 4 and Figure 5 show serotonin within-session variation using the same kind of aggregation. Inspection of these figures suggests an average increase in the values of oxytocin and serotonin between T0 and T1 during the majority of sessions of AAA compared to the CTRL group, in which a decrease is more frequent. Thus, figures suggest that this behavior may be related to treatment (VET1 vs. CTRL, and VET2 vs. CTRL, see Figure 3 and Figure 5) and to session week (Figure 2 and Figure 4).

Linear Mixed Effect (LME) analysis confirmed previous observations. In particular, we compared different mixed effects models, including (or not including) VET’ and ‘weeks’ as fixed effects and ‘subject’ as a random effect. From the comparison, it emerged that excluding one of the factors resulted in a significant difference (Likelihood Ratio Test, *p* < 0.05). Therefore, it could be inferred that the complete model, including both ‘VET’ and ‘weeks’, resulted in the best explanation of the data (Figures S3 and S4).

Furthermore, the results suggest that the first week showing a significant increase for both hormones was between the third and fifth week of the intervention (Figure 2 and Figure 4).

The effect of dog-zootherapist vet interaction and sessions was also observed: in particular, both dyads produced increases in oxytocin in the second half of the session cycle, starting from the sixth or seventh session. In addition, there were statistically significant effects in the within-session increase for both oxytocin and serotonin.

Patients who received the AAA interventions had a significant overall effect compared to the control group. Interestingly, we also noted an effect on the week: for oxytocin, the fifth and eleventh weeks had a significant average increase compared to the other weeks, while for serotonin, the first and eleventh weeks had a significant average increase compared to the other weeks.

Additionally, there was an interaction effect between the week and the zootherapist vet; for example, for oxytocin, dyad 2 produced a significant increase at week 5; several significant increases were produced by dyad 1 and dyad 2 starting at week 7.

Regarding serotonin, an interaction effect between the week and the vet was detected, e.g., dyad 2 produced a significant increase at week 3; the control groups had a significant decline at the fifth, sixth, and seventh weeks; dyad 1 and dyad 2 produced a significant increase at weeks 7 and weeks 11 and 12.

## 4. Discussion

Hemodialysis is one way to treat advanced kidney failure and can help individuals carry on an active life despite failing kidneys. Most people who require hemodialysis have different types of health problems, including the psychological aspect, which should not be overlooked [9,10,11,12,13]. Patients undergoing hemodialysis often experience increased levels of anxiety, pain, depression, stress, and adverse reactions related not only to the dialytic treatment itself but also to the awareness of living with a chronic disease [42]. Although pharmacological approaches are used to manage these psychopathological conditions, non-pharmacological techniques such as Music therapy, Cognitive Behavioral Therapy (CBT), and Intradialytic Exercise Training have also been widely demonstrated to improve the health-related quality of life of these patients [42,43,44]. Animal Assisted Activity (AAA) in a healthcare facility is an interdisciplinary approach involving animals as an adjunct to other conventional therapies for the treatment of certain types of patients [2]. As reported by Menna et al. [3], the interspecific relationship represents the focus and characterizing point of all AAIs, in which attachment styles play a primary role for set goals. An AAA in a healthcare facility represents a complex system of relational feedback between two different species, with the activation of bodily gestures, attitudes, and emotional sense–motor models, making this type of non-pharmacological approach unique.

Since data on the role of AAA intervention with the usual therapeutic activities performed in a hemodialysis center is scarce, this study aimed to examine the beneficial effects that AAA with a dog can bring to patients with renal disease during hemodialysis treatment, through the measurement of serum oxytocin and serotonin levels. In a previous study, performed in hemodialysis patients during a course of AAA with a dog by Menna et al. [45], a significant hormonal shift induced by the release of serotonin and oxytocin was demonstrated in dialysis patients. However, no significant difference was found between the start and end of the same session, no increases in serotonin and oxytocin were found after cessation of activities, and there was no control group.

In our study, the evaluation of the collected and processed data revealed statistical significance; the experimental group produced a statistically significant overall effect of an increase in both oxytocin and serotonin during the sessions compared to the control group. This investigation also highlighted the close relationship between the dog-vet dyad as a driving force for success through AAA sessions.

Indeed, this study revealed an effect due to the week and the interaction effect between the dog-veterinary zootherapist dyads (Figures S5 and S6).

Previous literature studies indicate how fundamental the interspecific relationship is in determining the result of safe interventions, which directly influence the well-being of the animal [46], and how much the relational features of the dog-veterinary zootherapist dyad influence the dog’s performance. A good attachment and a balanced and harmonious relationship between the dog and the veterinary zootherapist increase safety, reduce stress factors, and improve the dog’s performance [47]. These conditions manage to create a climate of trust and empathy that works as a basis for the emotional openness of patients, positively influencing the results of the intervention.

Furthermore, considering the characteristics and skills of the chosen animal means carrying out an AAI at its best. In this way, the well-being of the animal that is involved but never exploited is protected [46].

AAIs should be systematically evaluated as a network within a process where each component interacts and influences the other components [48].

According to this observation, the results achieved positively influenced the progress of the intervention, including the attention given to the relationship between the dog, the veterinary zootherapist, and the patients.

Furthermore, the results show that the fifth meeting represents the critical point of the AAA process, as in both veterinarians, there was a significant increase in both serotonin and oxytocin levels in the fifth week. This confirms the hypothesis that the fifth session could represent the step in which the therapeutic alliance is established, as reported in our studies [8,45,49].

A previous study examined the processes of change in three animal-assisted therapies (AATs) by assessing the relationship between human–animal bonding and therapeutic alliance, depth of processing, and uniformity of treatment sessions [49]. From the results that emerged, these parameters significantly improved during the sessions, confirming the thesis according to which the improvement of the therapeutic dimensions is a function of the human–animal bond. Therefore, high levels of the human–animal bond translate into an increase in the likelihood that the sessions have a greater therapeutic alliance and are experienced as deep and fluid.

Finally, while on the one hand this study has some strengths, indicated previously in the manuscript, it also has some weaknesses and shortcomings, which are as follows: the small size of sample patients and the limited number of the AAA sessions; the lack of knowledge of the cascade effects in the AAA of considered parameters (serotonin and oxytocin), which would be the key to understanding their role in mediating oxytocinergic functioning; involving a control group that receives a similar amount of attention from a researcher without a dog (an “attention control”) to discriminate the effect of the animal from the effect of novel social interaction; it has not been evaluated whether any eventual significant differences in the observed parameters exist between male and female patients; the lacked use of a questionnaire or a battery of psychological tests administered to the patients, to evaluate well-being and symptoms such as stress, fear, anxiety, depression.

## 5. Conclusions

Chronic kidney disease worldwide represents a major public health problem, associated with high levels of morbidity and mortality. People undergoing hemodialysis treatment are particularly exposed to the risk of a significant impairment of their quality of life, sometimes even developing conditions of real psychological suffering.

Therefore, it is important to take into consideration not only the clinical aspects of the patient but also the psychological ones, to improve survival and find a new adaptation and balance in the face of the disease.

To date, very little research is available regarding AAIs in patients undergoing hemodialysis treatment.

For this reason, in this work, we wanted to evaluate the potential benefits of a structured AAA intervention aimed mainly to improve the quality of life of patients during the dialysis cycle. Indeed, the emerging results showed that the AAA represents a positive stimulus for patients on dialysis, configuring itself as a non-pharmacological, complementary therapy to address the psychological burden of hemodialysis. Furthermore, considering the almost total lack of similar studies, it would be desirable to explore further aspects of the interspecific relationship useful for patients involved in AAA projects in the context of dialysis.

Finally, starting from our findings, it should be auspicious to promote future studies should larger sample sizes of patients, longer-term follow-ups, and direct comparisons with other psychosocial interventions and/or other non-pharmacological therapeutic approaches.

## Data Availability

The original contributions presented in this study are included in the article and Supplementary Material. Further inquiries can be directed to the corresponding author.

## Supplementary Materials

The following supporting information can be downloaded at: https://www.mdpi.com/article/10.3390/biomedicines13122944/s1. Figure S1. Oxytocin data. For each patient, within-session increases have been reported vs. week. Patient ID is reported in each panel title. The regression line has also been reported in each panel. The number of missing data is 34 (on 360 measures). Figure S2. Serotonin data. For each patient, a within-session increase has been reported vs. the week. Patient ID is reported in each panel title. The regression line has also been reported in each panel. The number of missing data is 34 (on 360 measures). Figure S3. Linear Mixed Effects Analysis of Repeated Measures for oxytocin data. Statistically significant effects in the within-session increase. The two VETS had a significant overall effect with respect to the control group. Their effects are not different. There was also an effect on the week: the fifth and eleventh weeks had a significant average within-session increase with respect to other weeks. Moreover, an interaction effect resulted between week and VET: e.g., VET2 produced a significant increase at the fifth week; many significant increases were produced by VET1 and VET2 since the seventh week. Figure S4. Linear Mixed Effects Analysis of Repeated Measures for serotonin data. Statistically significant effects in the within-session increase. The two VETS had a significant overall effect with respect to the control group. Their effects are not different. There was also an effect on the week: the first, second, tenth, and eleventh weeks had a significant average within-session increase with respect to other weeks. Moreover, an interaction effect resulted between week and VET: e.g., VET1 produced a significant increase at the fifth week; many significant increases were produced by VET1 and VET2 since the seventh week. Figure S5. Oxytocin data. For each patient, the distribution of within-session increases has been reported as a boxplot; each boxplot summarizes 12 weeks. Figure S6. Serotonin data. For each patient, the distribution of the within-session increase has been reported as a boxplot: each boxplot summarizes 12 weeks.

## Abbreviations

The abbreviations used in this manuscript are the following:

AAA	Animal Assisted Activities
AAIs	Animal Assisted Interventions
OXT	Oxytocin
SD	Standard Deviation
CTRL	Control
