# Supplementary material for: Animal Assisted Activities (AAAs) with Dogs in a Dialysis Center in Southern Italy: Evaluation of Serotonin and Oxytocin Values in Involved Patients
Source: Biomedicines. 2025 Nov 29;13(12):2944. doi: 10.3390/biomedicines13122944 (PMC12731044; doi:10.3390/biomedicines13122944)
Supplement: Supplementary file 1 [file biomedicines-13-02944-s001.zip › biomedicines-3933105-supplementary.pdf]

Supplementary Material

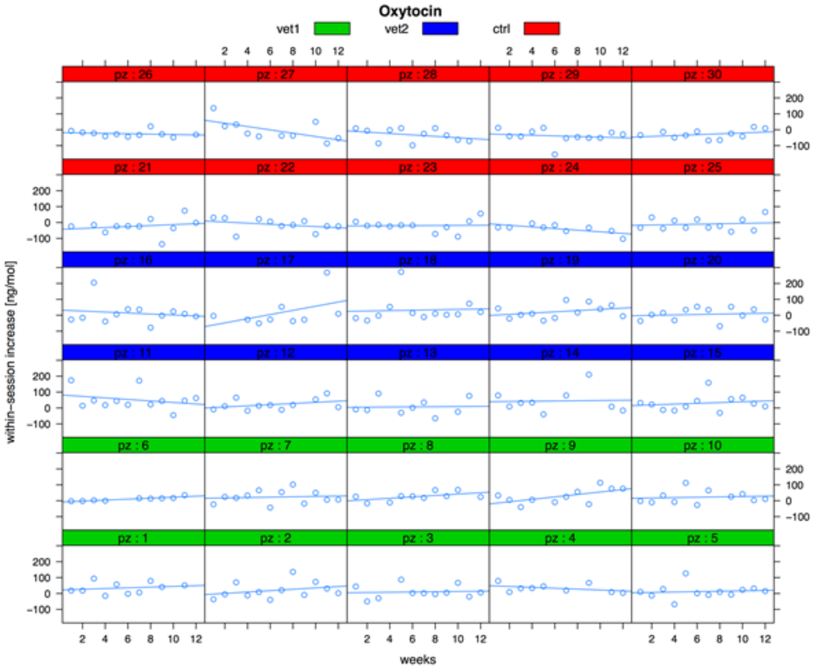

Figure S1. Oxytocin data. For each patient, within-session increases have been reported vs. week. Patient ID is reported in each panel title. The regression line has also been reported in each panel. The number of missing data is 34 (on 360 measures).

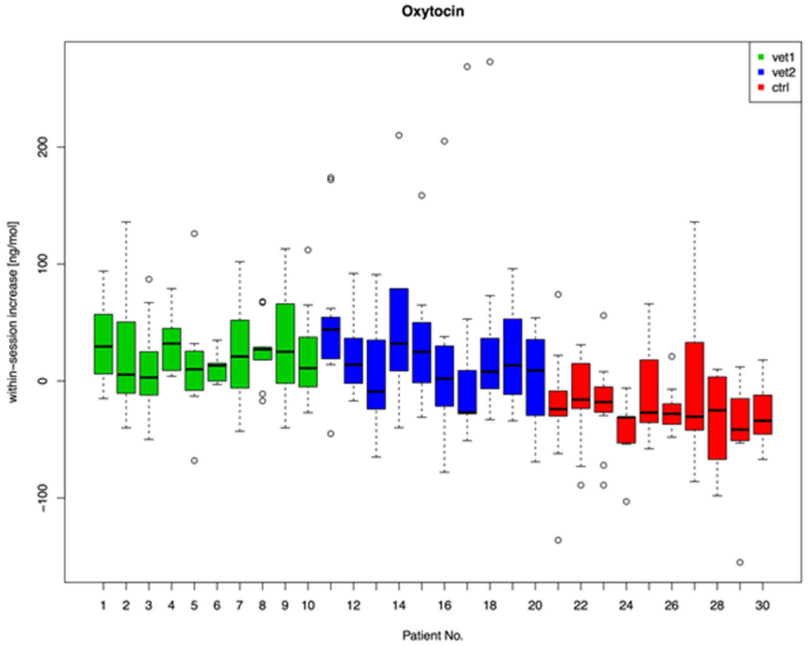

Figure S2. Oxytocin data. For each patient, the distribution of within-session increases has been reported as a boxplot; each boxplot summarizes 12 weeks.

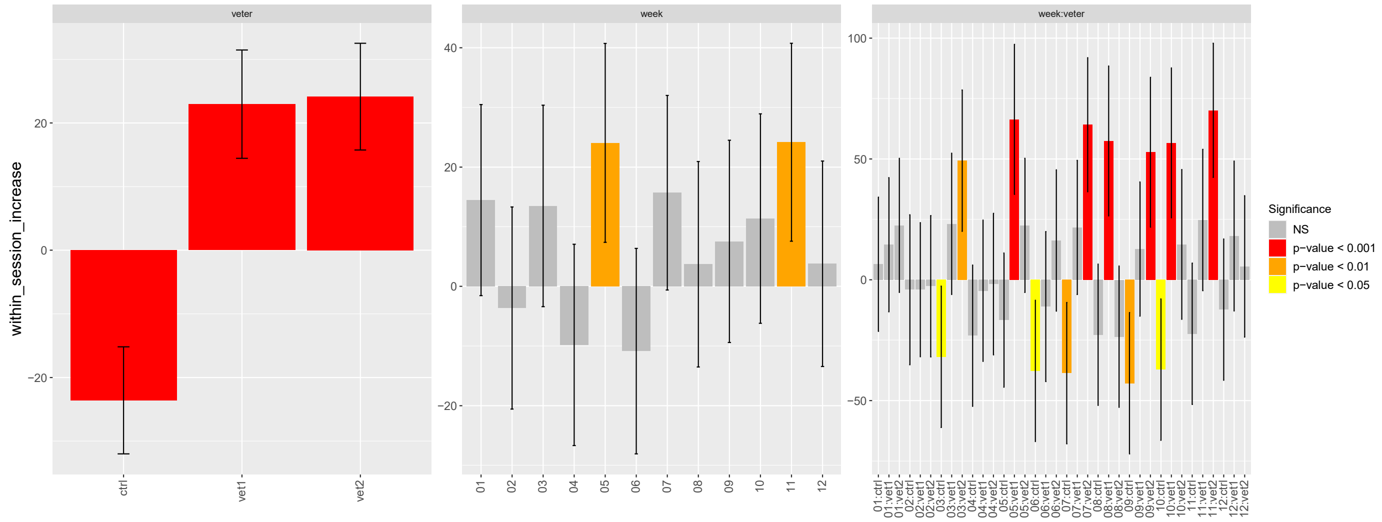

Figure S3. Linear Mixed Effects Analysis of Repeated Measures for oxytocin data. Statistically significant effects in the within-session increase. The two VETS had a significant overall effect with respect to the control group. Their effects are not different. There was also an effect on the week: the fifth and eleventh weeks had a significant average within-session increase with respect to other weeks. Moreover, an interaction effect resulted between week and VET: e.g., VET2 produced a significant increase at the fifth week; many significant increases were produced by VET1 and VET2 since the seventh week.

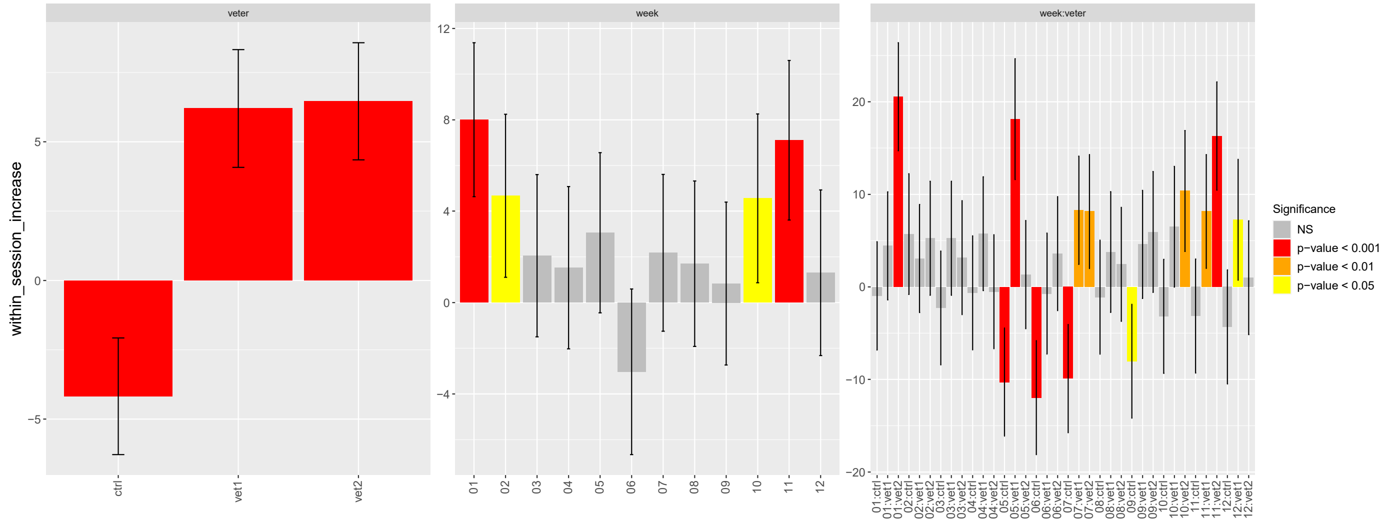

Figure S4. Linear Mixed Effects Analysis of Repeated Measures for serotonin data. Statistically significant effects in the within-session increase. The two VETS had a significant overall effect with respect to the control group. Their effects are not different. There was also an effect on the week: the first, second, tenth, and eleventh weeks had a significant average within-session increase with respect to other weeks. Moreover, an interaction effect resulted between week and VET: e.g., VET1 produced a significant increase at the fifth week; many significant increases were produced by VET1 and VET2 since the seventh week.

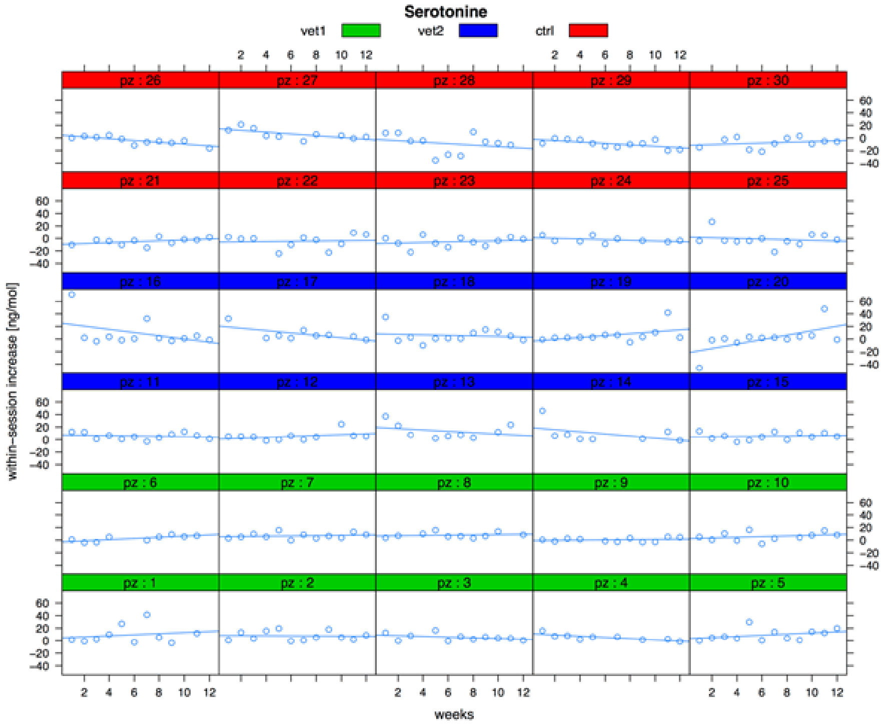

Figure S5. Serotonin data. For each patient, a within-session increase has been reported vs the week. Patient ID is reported in each panel title. The regression line has also been reported in each panel. The number of missing data is 34 (on 360 measures).

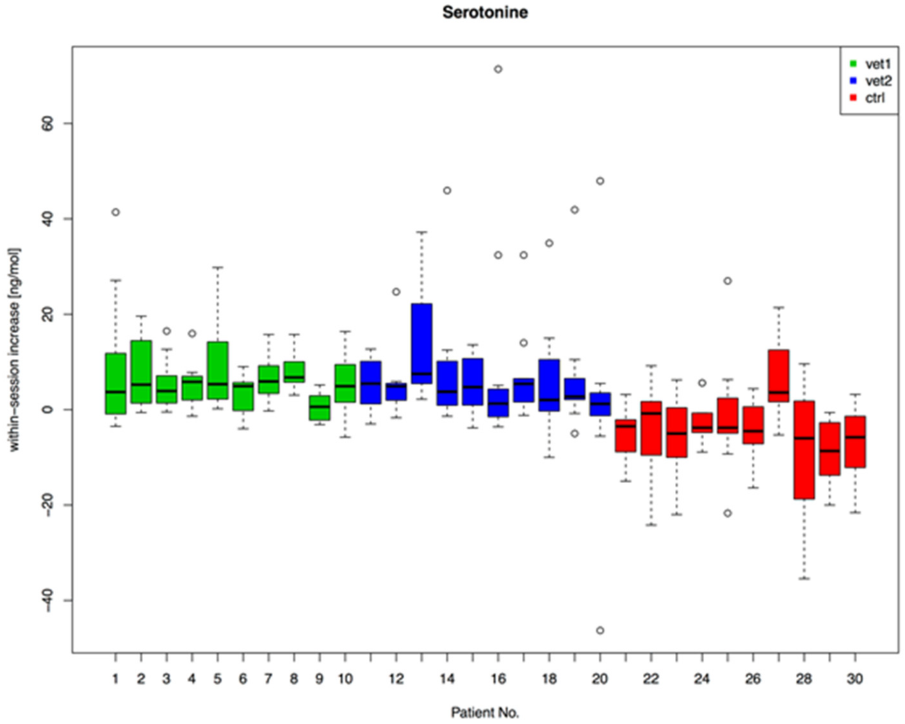

Figure S6. Serotonin data. For each patient, the distribution of the within-session increase has been reported as a boxplot: each boxplot summarizes 12 weeks.
